# Quarantine Convention of the South Atlantic and Gulf States, Held at Mobile, Ala., 1898

**Published:** 1898-03

**Authors:** 


### Society Notes.

### Quarantine Convention of the South Atlantic and Gulf States, Held at Mobile, Ala., 1898. Extract from Minutes.

The Committee on Resolutions submitted majority and minority reports.

The majority report favored the passage by Congress of the bill known as the "Spooner Bill."

The minority report requested Congress to enact a national measure of uniforn quarantine.

Hon. R. H. Clarke, of Alabama, offered a substitute, which was amended by the convention, and moved its adoption.

HON. R. H. CLARK'S AMENDED SUBSTITUTE.

*Resolved*, That it is the sense of this convention:

1. That Congress be requested to provide for a Department of Public Health as soon as practicable.

2. That Congress should enact laws to provide for an efficient maritime quarantine, to be uniform and impartial in its application to the different commercial ports of this country so as to give no one, or more of them, undue advantage over the others, to be enforced by the several States and municipal quarantine or health boards, if they will undertake so to do, leaving also to the States the power to prescribe and enforce additional reasonable safeguards of the health of their communities, provided that such State action shall not unreasonably obstruct commerce.

3. That Congress should aid the several States in establishing and maintaining uniform, reasonable and efficient quarantine laws for affecting, but not regulating, interstate commerce, leaving to each State adequate power to protect, as it shall deem best, the lives and health of its people.

4. That Congress should leave exclusively to the States the regulation of their purely internal commerce, and the provision of such quarantine and sanitary laws and regulations as they may deem advisable to that end.

5. That, in the framing of quarantine laws and regulations, and in their enforcement, Congress should avail itself of the learning, experience and ability of the medical profession in the fullest measure possible, and especially by way of an advisory council.

A full and free discussion ensued. * * *

The Chairman of the Committee on Resolutions then withdrew the majority report.

The minority report was then withdrawn by the gentleman who presented it, Mr. Kay, of Georgia.

The substitute was then submitted to the vote of the convention, and was unanimously adopted.

(Signed) C. P. WILKINSON, M. D., Chairman.
Address: Quarantine, La.

H. A. MOODY, M. D., Secretary.
Address: Mobile, Ala.

The following resolutions, adopted by the Quarantine Convention of the South Atlantic and Gulf States, are, by direction of said convention, forwarded to officers and members of the

Senate of the United States and of the House of Representatives:

TO ESTABLISH UNIFORMITY OF QUARANTINE RULES AND REGULATIONS IN CERTAIN STATES.

*Resolved*, That it is the sense of this convention of the States bordering on the South Atlantic and Gulf coast, viz.: Virginia, North Carolina, Georgia, Florida, Alabama, Mississippi, Louisiana and Texas, that they should, as soon as practicable, meet in conference and prepare a code of rules and regulations for the purpose of controlling and preventing the spread of yellow fever and other contagious or infectious diseases; said rules and regulations to be uniformly accepted and honored by the several health boards of the States mentioned; and further to adopt a system of pratique and health certificates to be used in times of epidemic, to be likewise honored by the several health boards of the States named.

A RESOLUTION.—TO ARRANGE FOR A CONVENTION OF HEALTH AND QUARANTINE OFFICIALS OF THE SOUTH ATLANTIC AND GULF COAST STATES TO ESTABLISH UNIFORM QUARANTINE RULES, REGULATIONS, ETC.

*Resolved*, That this convention call the proposed meeting of the health and quarantine officials of the South Atlantic and Gulf coast States to be held in Atlanta, Georgia, during the first week in April, 1898; and

*Resolved*, That the chairman of this convention appoint a Committee, to consist of one member from each State entitled to representation, whose duty it shall be to have charge of all necessary arrangements looking to hold the same.

The chairman appointed the following committee in compliance with the above resolution: Hon. C. A. Collier, of Georgia; Dr. H. A. Moody, of Alabama; Dr. F. G. Renshaw, of Florida. Dr. H. B. Horlbeck, of South Carolina; Prof. J. L. Ludlow, of North Carolina; Dr. Bat Smith, of Texas; Dr. Felix Formento, of Louisiana; Dr. E. F. Griffin, of Mississippi.

A RESOLUTION LOOKING TOWARD THE SANITARY INSPECTION AND SANITATION OF FOREIGN PORTS THAT MENACE US MOST, AND TO THE ESTABLISHMENT OF A SYSTEM OF INTERNATIONAL QUARANTINE.

*Resolved*, 1st. That the Congress of the United States be requested to authorize the President to take such steps, by treaty or otherwise, as may aid in inducing the respective governments of the Inter-Tropical American ports to secure proper and adequate sanitation; together with the adoption by them of such restrictive measures as may be necessary to render such ports in good sanitary condition and to prevent the introduction of yellow fever.

2d. To provide for the maintenance of a medical force in this country in each such port, to give warning of the existence of yellow fever therein, with adequate power for the most efficient possible prevention of the communication of the disease therefrom; and that Congress be memorialized to make such appropriation as may be necessary to maintain a proper medical inspection service in Inter-Tropical American ports of sufficient importance to warrant such appointment.

3d. That the Congress of the United States be memorialized to make a suitable appropriation and provide for the early calling of a conference of port sanitary authorities to deal with the subject of International Quarantine and Preventive Sanitary regulations. Respectfully submitted by

JOHN B. HAMILTON,
S. R. OLLIPHANT,
H. R. CARTER,
R. P. DANIEL,
P. J. HAMILTON,
Committee.

### A RESOLUTION LOOKING TOWARD THE SCIENTIFIC INVESTIGATION OF THE CAUSE AND PREVENTION OF YELLOW FEVER.

*Resolved*, That the convention respectfully recommend to the Congress of the United States that the following bill be passed:

*Be it enacted by the Senate and House of Representatives of the United States of America in Congress Assembled:*

SECTION 1. A commission of experts shall be appointed by the President for the purpose of making investigations relating to the cause and prevention of yellow fever.

SEC. 2. This committee shall consist of four expert bacteriologists, one to be detailed from among the medical officers of the army, one from among the medical officers of the navy, one from among the medical officers of the Marine Hospital Service, and one to be appointed from civil life.

SEC. 3. The President shall name one of the members of the commission as chairman, who shall direct the work of the commission and report to him from time to time the results attained. The headquarters of the commission shall be in Washington, D. C., and one or more members shall be detailed to conduct investigations in the city of Havana, Cuba, or in some other locality where yellow fever prevails.

SEC. 4. The medical officers of the army, the navy and the Marine Hospital Service detailed as members of this commission shall receive no compensation beyond their salaries. But during the time that they are necessarily absent from their proper stations in the performance of the duties imposed upon them by this act, their necessary living and traveling expenses shall be paid from the appropriation made in this act. The civilian member of the commission shall receive, in addition to his necessary living and traveling expenses, six dollars per day during the time he is actually employed in prosecuting the investigation contemplated by this act.

SEC. 5. The sum of twenty thousand dollars is hereby appropriated out of any money in the treasury of the United States not otherwise appropriated, for carrying out the provisions of this act, and for the purchase of necessary instruments and material, for rent of a laboratory in Havana, Cuba, or elsewhere, and for other incidental expenses.

A RESOLUTION CONCERNING SENATE BILL NO. 1063.

WHEREAS, We are informed that a bill is now pending in the Senate of the United States, known as Senate Bill No. 1063, to regulate the practice of vivisection in the District of Columbia; and

WHEREAS, By its enactment we feel convinced that the progress of scientific knowledge, especially in the direction of the prevention of disease, would be limited and restricted, if not altogether prevented; therefore be it

*Resolved.* That it is the sense of this convention that the passage of the said Senate Bill No. 1063 would be unwise, injurious and prejudicial to the interest of Sanitary science, and that the officers of this convention notify the Honorable the Senate of the United States accordingly.

A RESOLUTION DIRECTING COPIES OF RESOLUTIONS TO BE FORWARDED TO THE CONGRESS OF THE UNITED STATES.

*Resolved*, That the secretary of this convention shall prepare and forward to the members of Congress of the South Atlantic and Gulf States copies of the resolutions this day adopted, duly certified, with the signatures of the officers of this convention, and shall request the aforesaid members of Congress to bring the subject matter to the attention of their respective houses; and to prepare and introduce the requisite bills to effect the purpose of the resolutions, and to procure the passage thereof.

We, the undersigned, certify that the foregoing are correct copies of the resolutions as adopted by the convention.

(Signed) C. P. WILKINSON, M. D., Chairman.
H. A. MOODY, Secretary. Address: Quarantine, La.
Address: Mobile, Ala.

